# Recognition of natural objects in the archerfish

**DOI:** 10.1242/jeb.243237

**Published:** 2022-02-10

**Authors:** Svetlana Volotsky, Ohad Ben-Shahar, Opher Donchin, Ronen Segev

**Affiliations:** 1 Department of Biomedical Engineering, Ben-Gurion University of the Negev, Be'er Sheva, 8410501, Israel; 2 Zlotowski Center for Neuroscience, Ben-Gurion University of the Negev, Be'er Sheva, 8410501, Israel; 3 Department of Computer Science, Ben-Gurion University of the Negev, Be'er Sheva, 8410501, Israel; 4 Department of Life Sciences, Ben-Gurion University of the Negev, Be'er Sheva, 8410501, Israel

**Keywords:** Visual object recognition, Object categorization, Computational model, Visual system

## Abstract

Recognition of individual objects and their categorization is a complex computational task. Nevertheless, visual systems can perform this task in a rapid and accurate manner. Humans and other animals can efficiently recognize objects despite countless variations in their projection on the retina due to different viewing angles, distance, illumination conditions and other parameters. To gain a better understanding of the recognition process in teleosts, we explored it in archerfish, a species that hunts by shooting a jet of water at aerial targets and thus can benefit from ecologically relevant recognition of natural objects. We found that archerfish not only can categorize objects into relevant classes but also can do so for novel objects, and additionally they can recognize an individual object presented under different conditions. To understand the mechanisms underlying this capability, we developed a computational model based on object features and a machine learning classifier. The analysis of the model revealed that a small number of features was sufficient for categorization, and the fish were more sensitive to object contours than textures. We tested these predictions in additional behavioral experiments and validated them. Our findings suggest the existence of a complex visual process in the archerfish visual system that enables object recognition and categorization.

## Abstract (precis)

**Highlighted Article:** Archerfish are capable of natural object recognition and categorization based on a small number of visual features.

## INTRODUCTION

**Fig. 1.**
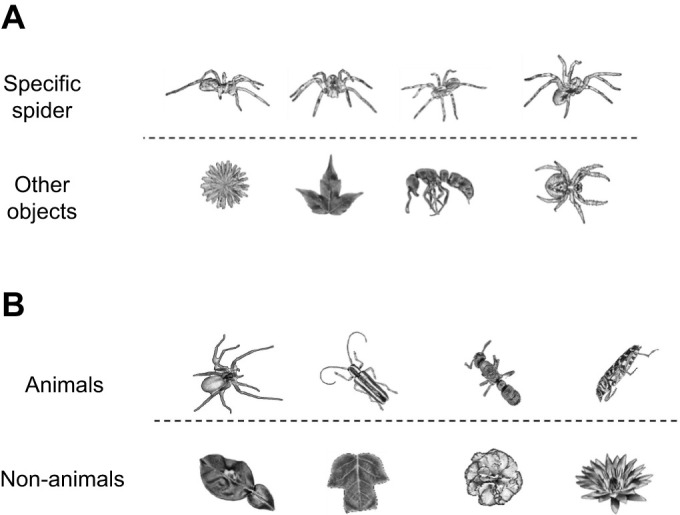
**Object recognition problem.** Object recognition involves the identification of objects regardless of transformations in size, contrast, orientation or viewing angle. (A) An example of object recognition. In this case, a specific spider needs to be identified in the presence of other insects. (B) An example of object recognition of an object class. In this case, an animal (insect or spider) needs to be recognized in the presence of non-animate objects (leaves or flowers).

For their survival, many animal species require the computational capacity to perform a range of complex object recognition tasks, from identifying a conspecific to recognizing a camouflaged predator, and classifying an item as edible (DiCarlo et al., 2012; Santos et al., 2001; Suboski and Templeton, 1989). Object recognition is defined as the ability to rapidly and accurately identify a specific object (Fig. 1A) or categorize objects into classes (Fig. 1B) despite substantial differences in the retinal representation of the object or across category members (DiCarlo et al., 2012). Variations in an object's retinal image are typically caused by the different conditions under which the object is viewed; for example, its illumination, the viewing distance and angle, and other environmental characteristics (Biederman and Bar, 1999; DiCarlo and Cox, 2007; DiCarlo et al., 2012). The ability of animal brains to recognize objects in an efficient and accurate manner depends on powerful neural computations that enable classification and identification.

Although there is convincing evidence that animals outside of the mammalian clade are capable of object recognition, the mechanisms and formal algorithms underlying this performance remain poorly understood. Pigeons, for example, are capable of categorizing natural objects, human faces and even emotional expressions (Soto and Wasserman, 2014; Watanabe et al., 2019). Similarly, bees (Avargues-Weber et al., 2010; Giurfa et al., 1997; Werner et al., 2016) and wasps have all been shown to be capable of conspecific visual identification.

A number of studies have indicated that fish also have the capacity to differentiate between different shapes (Lucon-Xiccato et al., 2019; Mackintosh and Sutherland, 1963; May et al., 2016; Oliveira et al., 2015; Siebeck et al., 2009) and intensities (Agrillo et al., 2016), use hierarchy of visual processing in discriminating shapes (Truppa et al., 2010) and fish faces (Parker et al., 2020), and the archerfish can even be trained to discriminate between human faces (Newport et al., 2016, 2018). For simple abstract stimuli, fish are capable of overcoming changes in orientation (DeLong et al., 2018), size consistency (Douglas et al., 1988) and the occlusion of objects (Sovrano and Bisazza, 2008). In addition to these findings in teleosts, there is an interesting study in sharks, showing they were able to discriminate teleost fish from snails (Schluessel and Düngen, 2015).

To extend our understanding of object recognition in teleosts and non-mammalian vertebrates, we examined the recognition of natural objects in the archerfish (*Toxotes chatareus*). The rationale for selecting the archerfish draws, in part, on the potential benefits of studying organisms distant from mammals on the evolutionary scale (Karoubi et al., 2016), as this may point to additional visual mechanisms or basic principles. At the same time, the value of the archerfish as an animal model stems from the fact that these fish can be trained to discriminate stimuli visually by shooting at the selected option. This is the case even when images are presented on a computer screen, and the fish's choice can be reported in a simple experimental manner similar to that used for birds and primates, which report the decision by pressing the screen (Ben-Simon et al., 2012a,b; Ben-Tov et al., 2015, 2018; Gabay et al., 2013; Mokeichev et al., 2010; Newport et al., 2013, 2014, 2015; Newport, 2021; Reichenthal et al., 2019; Schuster et al., 2004; Schuster, 2007; Vasserman et al., 2010). By utilizing this fish's remarkable ability to shoot down insects and other small animals that settle on the foliage above the water line with a jet of water from the mouth (Lüling, 1963), these fish can be trained to perform an object recognition task and essentially report their decisions using stimuli in the lab. Thus, the archerfish can provide the fish equivalent of a discriminative response by a monkey or a human when performing a recognition task with a click of a button. Finally, using a support vector machine (SVM) classifier together with features derived from the objects in the behavioral experiment, we analyzed the visual features the fish is using in categorization. We tested the predictions in complementary experiments and found them to be consistent with the fish behavior.

## MATERIALS AND METHODS

### Animals

Fourteen archerfish, *Toxotes chatareus* (Hamilton 1822), subjects participated in the experiments. Usage of fish in each experiment is described below. Adult fish (6–14 cm length, 10–18 g mass) were purchased from a local supplier. The fish were kept separately in 100 l aquaria filled with brackish water at 25–29°C on a 12 h:12 h light:dark cycle. Fish care and experimental procedures were approved by the Ben-Gurion University of the Negev Institutional Animal Care and Use Committee and were in accordance with the government regulations of the State of Israel.

### Training

After a period of acclimation to the laboratory conditions, new water tank, food and temperature, inexperienced fish were gradually trained to shoot at targets presented on a computer screen (VW2245-T, 21.5 inch, BenQ) situated 35±2 cm above the water level. Each training session consisted of 20 trials and was conducted 2–3 times a week. In the first stage, the naive fish were trained to shoot at a single black circle on a white background that appeared at random locations on the screen. A blinking black square appeared immediately prior to the display of the target in the middle of the screen and was used as a cue to draw the fish's attention upward. If the fish shot at the target within 15 s of its appearance, it was rewarded with a food pellet. Otherwise, the target disappeared and the next training trial started. The mean response time of the fish ranged from 2 to 10 s. The training continued until the fish succeeded in hitting 80% of the targets within 15 s.

**Fig. 2.**
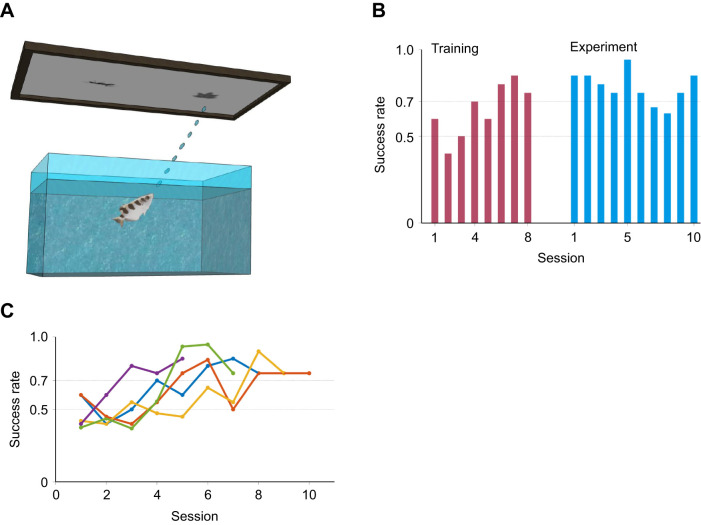
**Behavioral experimental setup.** (A) The archerfish is presented with two objects on the screen: a target and a distractor. The fish is rewarded if it selects the target image. (B) An example of success rate per session in the training process (red) and experiment (blue) of fish 1 in the spider recognition task (20 trials each session). (C) Learning curve of five example fish. The fish reached 70% success rate or higher in three successive sessions after 5–10 sessions of training.

After the fish learned to shoot at targets on the screen, they were trained to recognize either a specific object or a category through a two-alternative non-forced choice procedure (Fig. 2A). A session of 20 trials where the fish had to choose and shoot at one of two images was repeated over several sessions to familiarize the fish with the experiment. When the fish achieved a 70% success rate in three consecutive sessions in choosing the designated object or category, it was considered trained and ready for the experiment (examples of the training and experimental procedure for one fish in a spider recognition task are shown in Fig. 2B). The training phase usually took 5–10 sessions, as can be seen in Fig. 2C for five fish. The subsequent experimental trials were recorded and these results were used for the analyses.

### Stimuli

For all experiments, we used images of objects familiar to the fish from their natural environment. The images were composed of edible and inedible objects (from the archerfish's perspective): the inedible objects were either leaves or flowers, whereas the edible objects were either spiders or insects such as a cockroach, an ant or a beetle. The images of the insects and the spiders were obtained from the BugwoodImages project website (insectimages.org). Images of flowers were taken from the Oxford Flowers 102 dataset (Nilsback and Zisserman, 2008), and images of leaves were taken from the Flavia Plant Leaf Recognition project dataset (Wu et al., 2007). Multiple shots of one specific spider and one ant were taken from the animated 3D models. The models were purchased from the Sketchfab store under standard license (sketchfab.com).

All images were preprocessed using Matlab. All background colors were removed and the objects, after being converted to grayscale, were placed on a white background. The motivation for removing color was to reduce the number of dimensions in the visual stimuli. In a pilot study, we found that the fish perform with and without color with similar success rate. Thus, for simplicity of the analysis, color was removed. The size of the objects was randomized in the following way: the number of the pixels in the image was selected to have a uniform distribution from a discrete set of object sizes. For this purpose, the images were resized to create 5 levels of object area, defined as the number of pixels within the contour of the object: ∼10,000 pixels, ∼50,000 pixels, 100,000 pixels, 200,000 pixels and 300,000 pixels.

### Experiment 1

We investigated recognition of a specific object in the archerfish. A total of four fish were used in this experiment. The fish were rewarded with a food pellet if they selected the target. The experiment consisted of 10 sessions with 20 trials per session, and lasted 4–5 weeks with 2–3 sessions per week. This selection of experimental parameters was chosen to avoid overfeeding and loss of motivation by the fish. We did not observe a decline in success rate over time. Overall, we used 400 images with 200 pairs of images presented in all trials. Fish responded in 75–99% of all trials.

There were two types of targets. First, an image of a specific spider was presented to the fish together with a distracting object. The distracting objects were leaves, flowers, insects or other spiders. The target spider was shown from different viewpoints, with different orientation, size, contrast and screen locations. All presentation parameters were randomized. Out of 200 pairs of images, 75% were of the target spider presented together with different objects, and 25% were of the target spider presented together with other spiders.

Then, the experiment was repeated with a designated target of a specific ant. The distractors in this case were images of leaves, flowers, insects or other ants.

### Experiment 2

Whereas experiment 1 focused on specific objects (and thus on identification), experiment 2 explored the ability of the archerfish to generalize and categorize various objects into classes in a superordinate-level categorization (Soto and Wasserman, 2014). A total of 10 fish were used in this experiment. In each trial, two novel images belonging to two different categories – edible and inedible – were presented in random locations on the screen. Overall, we used 1640 images with 820 pairs of images presented in all trials. Fish responded in 86–99% of trials.

### Analysis of image features

To analyze the possible visual features that may help the fish perform object recognition, we used a model based on two main stages of computation. The first is extraction of 18 features from each stimulus image, followed by a categorization of this feature vector by machine learning classifier. Adding or removing features from the analysis can reveal which features might be used by the fish in the process. Second, we used validation experiments to find out whether these features are indeed used by the fish.

We extracted a set of 18 visual features commonly used in image processing from each image (Nixon and Aguado, 2019; Wang et al., 2012; Wen et al., 2009) and then used these features to characterize the images. The following features were used. (1) Object area, defined as the number of pixels within the object's perimeter. Features describing object compactness: (2) convex hull area; (3) convex hull area divided by the object area; (4) perimeter length; (5) roundness, defined as the perimeter squared divided by 4π×area. Features describing object curvatures: (6) the number of sharp curves in the object's perimeter, defined as follows. First, the object's perimeter was divided into sections with a length of 100 pixels each; then, a second degree polynomial was fitted to each section, and the polynomial's second derivative was used as the curvature for every section; finally, a section with curvature values above the standard deviation of all values for all sections was considered a sharp curve, which yielded the number of sharp curves in the object's perimeter. (7) Average curvature value of the sections with sharp curves as defined in 6. Features describing object shape eccentricity: (8) Shape eccentricity, defined as the ratio between the foci of the ellipse that surrounds the object and the length of its major axis; (9) convex hull eccentricity, defined as the ratio between the foci of the ellipse that surrounds the convex hull of an object and the length of its major axis. Features describing object texture: (10) entropy of light intensities of the object’s pixel values; (11) standard deviation of the object's pixel values; (12) skewness, defined as the normalized third central moment of the object's pixel value distribution (13) correlation between the object and a checkerboard: dot product of the object with 5 checkerboards with different checker sizes – 4–12 checkers in a row – where the maximum result was used. Other features: (14) correlation between the object and a star: dot product with a star shape to measure the resemblance of the object to a star; (15) symmetry, defined as the distance between two halves of an image on the horizontal and vertical axis of the image; all images were rotated to align the major axis of the surrounding ellipse to the *x*-axis; (16) symmetry defined as the Euclidean distance between two halves of the image on the horizontal and vertical axis of the image silhouette. Image energy: (17) mean image energy defined as the average value of all pixels in the image; (18) total image energy, defined as the sum of all pixel values in the image.

### SVM analysis

We used Matlab Statistics and the Machine Learning toolbox functions to build a SVM classifier. The classifier was trained using a matrix with image features and the fish's responses as labels. The number of images used in each model ranged from 1396 to 1620, depending on the number of trials that the fish responded to. The training set consisted of a random 75% of the images. The resulting SVM model was tested on the remaining 25% of the images. The labels that the model returned were compared with the images' true labels and with the fish's behavioral selection. The average success rate for 20 iterations was used as the model's success rate. We arranged the features according to the order of their contribution to the model using a greedy algorithm. Specifically, the features are added to the SVM model one by one, in the order of their contribution: first, a model is built with each feature separately and the one with highest success rate in predicting a category is the first feature added to a model; then, the model is run with two features: the first one and all the rest – one by one, the feature that resulted in the highest success rate is added. Thus, at every step, the feature that contributed the most to the model's success was added.

### Statistical analysis

We performed a hierarchical Bayesian analysis to evaluate the behavior of each fish in every experiment. The statistical analysis was done with the R 4.0.4 programming language (https://www.r-project.org/) and the JAGS 4.3.0 statistical package (https://mcmc-jags.sourceforge.io/), (Plummer, 2003). We used these together in a Bayesian data analysis that produced samples from the probability distribution of the parameters of interest given the data (Kruschke, 2014). The central parameters of interest were the success rates for each fish *p*_fish_. These parameters described the data through a binomial distribution:
(1)

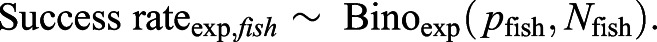
The *p*_fish_ themselves were assumed to come from a beta distribution:
(2)

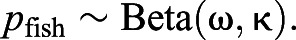
Beta distributions are a very general family of distributions in the range between 0 and 1. We parameterized the beta distribution by the mode, ω, and concentration, κ. ω is a parameter that describes the success rate which is most likely for a fish. κ describes how broadly spread out the fish's success rate is around ω. The priors for ω and κ were chosen to be uniform and very broad.

Bayesian data analysis takes data and a model and produces samples of possible values of the parameters of the model consistent with the data. This procedure provides the posterior distribution of the parameters given the data. The algorithm most commonly used to generate these samples is the Markov Chain Monte Carlo (MCMC) algorithm. We generated 3 chains of 10,000 MCMC samples from the joint posterior probability distribution of all the parameters (*p*_fish_ for all fish, ω and κ) for each experiment. The standard procedure is to use 3 or 4 chains to show that they converge to a similar result to verify robustness of the outcome. Convergence of the algorithm and sampling properties were tested using the graphical and quantitative methods outlined in Kruschke (2014). Using the MCMC samples, we calculated the 95% highest density interval (HDI) for the fish's behavioral success rate, a range of values in which there was a 95% posterior probability of finding the parameter. The success rate of the experiment was considered significantly above the chance level if 95% HDI of its posterior distribution was greater than a region of practical equivalence (ROPE) of 5% around the chance level of 50%. Similarly, if 95% HDI of the difference in success rate between the two experiments included the ROPE of 5% around zero, the success rate was not considered to be different.

## RESULTS

We characterized the archerfish's ability and processing during an ecologically relevant object recognition task. For this purpose, we conducted two alternative non-forced choice experiments using a continuous reinforcement schedule for correct responses. Generally, the fish was presented with images of natural stimuli that are important in the fish habitat, and was rewarded for an appropriate shooting response. The two stimuli were presented simultaneously on a computer monitor situated above the water tank (Fig. 2A). A shooting response directed at the correct target was rewarded with a food pellet, whereas selection of the other stimulus was not. An incorrect response resulted in termination of the trial and moving to the next one. Successive learning trials were contingent on the fish collecting the reward for the previous correct response. To neutralize the effect of position bias in the fish's responses, the two targets were presented at a random location on the screen.

### Archerfish can recognize specific objects regardless of differences in contrast, size and viewing angle

**Fig. 3.**
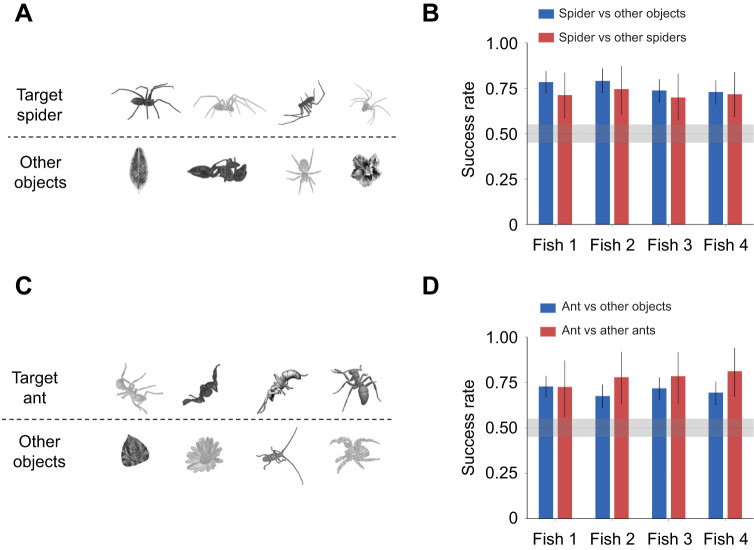
**Archerfish are capable of invariant object recognition.** (A) Examples of a single target spider from different viewpoints and with different contrast levels (top row) and other distractor objects and spiders (bottom row). (B) Success rate of three archerfish in recognizing the target spider: means±95% highest density interval (HDI). Shading around the chance level of 0.5 represents a region of practical equivalence (ROPE) of 0.05. Fish responded in 149–199 trials in each experiment (out of 200 trials). (C) Examples of a single target ant from different viewpoints and with different contrast levels (top row) and other distractor objects and ants (bottom row). (D) Success rate of three archerfish in recognizing the target ant: means±95% HDI. Shading represents a ROPE of 0.05 around the chance level of 0.5. Fish responded in 182–200 trials in each experiment (out of 200 trials).

We tested whether archerfish are capable of object recognition. Three archerfish were trained on a set of pictures of a single spider viewed from different angles in 3D space and then tested on the same spider viewed from other angles (the generalization set), which were not included in the original set (Fig. 3A). The target spider was presented under different conditions such that size, viewing angle, contrast and location varied from trial to trial. The target spider was presented together with another object that could be a leaf, a flower, an insect or another spider (Fig. 3A; see Materials and Methods).

The fish were able to recognize and choose the target spider, both on trials where the second object was not a spider and also against other spiders (Fig. 3B). For all fish in both experiments, success rate was significantly higher than the chance level. Even for the fish with lowest success rate, 95% HDI ranged from 0.66 to 0.79, which is higher than a ROPE 5% around chance level of 0.5 (see Materials and Methods, ‘Statistical analysis’). For the best performing fish, 95% HDI range was 0.72–0.84. In addition, the 95% HDI of the difference between the success rate of individual fish on trials with two spiders and the trials with a spider and non-spider completely contained a ROPE of 5% around chance level, indicating that the fish could differentiate the target spider from other types of spiders as well as from other objects.

A similar experiment was conducted with an ant as the target image. The same three fish were retrained to recognize one specific ant that was shown together with other objects, and sometimes with other ants (Fig. 3C). The fish learned to differentiate the target ant from the other objects and also from other ants (Fig. 3D). The success rates in this experiment were not significantly different from the rates in the experiment with a spider target.

### Archerfish can categorize objects into classes and learn to generalize from examples

**Fig. 4.**
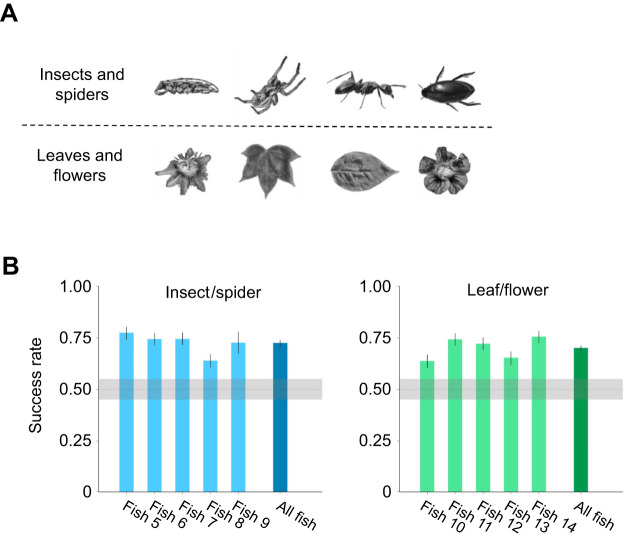
**Archerfish can categorize novel objects into groups.** The fish were trained to categorize animal and non-animal objects. (A) Examples of animal objects (insects and spiders, top row) and non-animal objects (leaves and flowers, bottom row). (B) Success rate of 10 fish in selecting an object from its designated category: means±95% HDI. Shading represents a ROPE of 0.05 around the chance level of 0.5. Fish 5–9 were rewarded for choosing an animal; fish 10–14 were rewarded for choosing a non-animal. Fish responded in 694–814 trials (out of 820 trials).

We tested the ability of the fish to discriminate between the images of two categories of stimuli (Fig. 4A): non-animals (leaves and flowers) and animals (spiders and insects comprising ants, beetles and cockroaches) in a superordinate-level categorization (Soto and Wasserman, 2014). In this two-alternative choice task, in the first stage of the experiment, the animal category was rewarded and the non-animal category was not rewarded. The images were grayscale, normalized to five different sizes, shown at different locations on the screen and were never repeated; that is, each image was used only once (around 1600 images in total were used in the experiments). After 3–8 sessions, the success rate of the fish reached a plateau that was significantly above chance level (Fig. 4B). For the fish with lowest success rate, 95% HDI ranged from 0.61 to 0.67, which is higher than a 5% ROPE around chance level of 0.5. For the best performing fish, 95% HDI range was 0.74–0.80.

To test whether archerfish are predisposed to shooting at animals rather than plants, we tested four additional fish, which were trained to shoot at the non-animal targets (i.e. non-edible). Again, we found that the archerfish were able to select the non-animal targets at a significantly higher level than chance (Fig. 4B). This is an indication that the archerfish is not hardwired to select an animal.

### Archerfish can use five complex visual features to perform object recognition

**Fig. 5.**
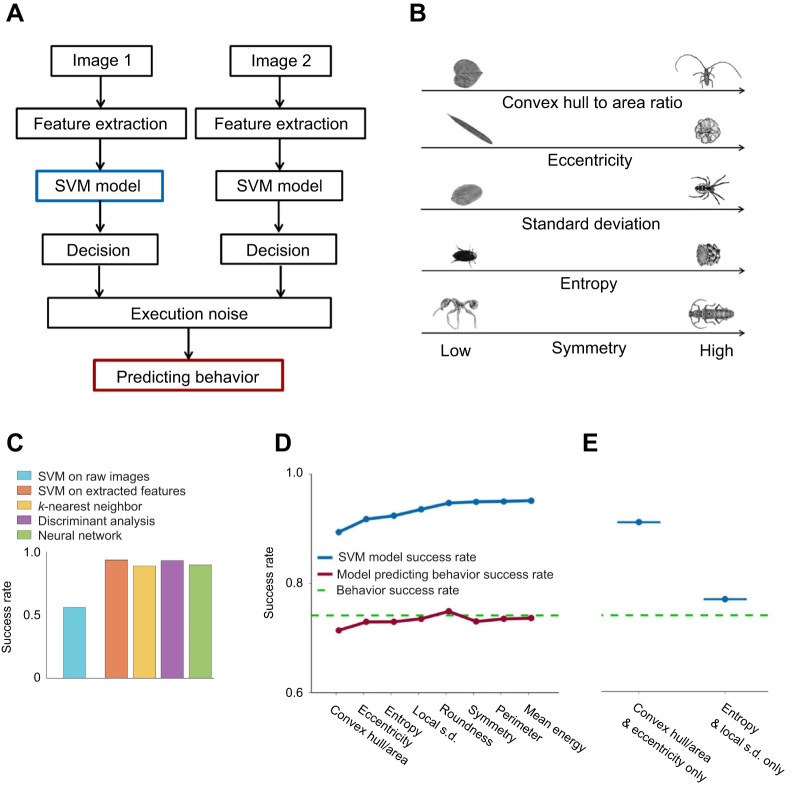
**Model building.** (A) In a behavioral experiment, the fish was exposed to two objects, made a decision about the object category and executed a shot. The support vector machine (SVM) classifier was fed with the features extracted from the images. (B) Examples of the extracted visual features. (C) Success rate of different classifiers: SVM classifier using raw images, SVM classifier using extracted features, *k*-nearest neighbor, discriminant analysis and neural network. (D) SVM classifier success rate in predicting the objects' true category (blue line, which corresponds to the blue outlined box in the model) and the model's success rate in predicting fish selection (red line, which corresponds to the red outlined box in the model). Separate features were added in the order of their contribution to the classifier's success. (E) SVM classifier success rate for combinations of features: two features of shape and two features of texture.

To identify the visual features used in the behavioral task, we built a model that simulated the process of object selection in the fish and fitted the model to the response data we collected on fish target selection. The model was composed of two branches of information processing, each processing one stimulus image in parallel (Fig. 5A). Each image recognition module was composed of a feature extraction stage followed by a classifier. The result of the computation by each module was fed into a decision module which, after adding execution noise, led to the behavioral choice of the model. The decision was made by comparing the classification output and verifying its consistency. If the classifications were different, the decision followed suit to the desired class. If the two classifiers returned the same category, the decision was made randomly by sampling a Bernoulli distribution with 0.5 probability of success. The behavioral noise itself was added to the decision vector: a decision response was flipped for pairs of images with a probability matched for each fish separately – from 0.65 to 0.8 – to get a success rate fit for the behavioral result.

For the classification module, we used a SVM classifier. The SVM was fed by visual features extracted from each image (examples in Fig. 5B; see Materials and Methods). We extracted a set of 18 features from each image and then used the SVM to build a classifier based on the fish's responses to the targets and on the extracted features. The features were selected heuristically for the image set (see Materials and Methods).

We compared the performance of the SVM classifier trained on the raw images with that of a classifier trained on a feature matrix and found that the use of features significantly improved performance. We also tried classifiers other than the SVM. There was no significant difference in their performance, so we continued with the SVM and features for the remainder of the analysis (Fig. 5C).

The classifier was built in an iterative manner, starting with the most informative feature, i.e. the feature with the highest success rate when used in the model separately, then adding the next most informative feature and so on, until the predictive value of the model became saturated. We used a standard training set, verification set and test set to avoid over-fitting the model. Although this was a greedy algorithm that could not guarantee an optimal solution, it still provided a lower bound for the optimal performance.

To test the model (Fig. 5A), we used it to simulate the behavioral experiment. The recognition rate at the output stage of the model matched the behavioral success rate of the fish (Fig. 5D), indicating the capability of the model to capture the statistics of fish behavior. Next, we analyzed the model structure to reveal aspects of the fish's decisions.

### Shape is more important than texture in archerfish object recognition

Fig. 5D shows that using only the first five features that describe an object's shape compactness (ratio of convex hull to area and roundness), shape eccentricity and texture (entropy and the local standard deviation), the model's success rate saturated. Using these five features, the model achieved a success rate of 94% compared with a success rate of 95% on all 18 features.

We calculated the model's success rate given only the first two shape features; specifically, the ratio of the convex hull to the area together with eccentricity. The model's predictions were close to saturation, with a success rate of 92% (Fig. 5E). When given only the two most important texture features, entropy and the local standard deviation, the model's success rate was only 76%. This suggests that shape was more important than texture in the visual discrimination performed by these fish.

**Fig. 6.**
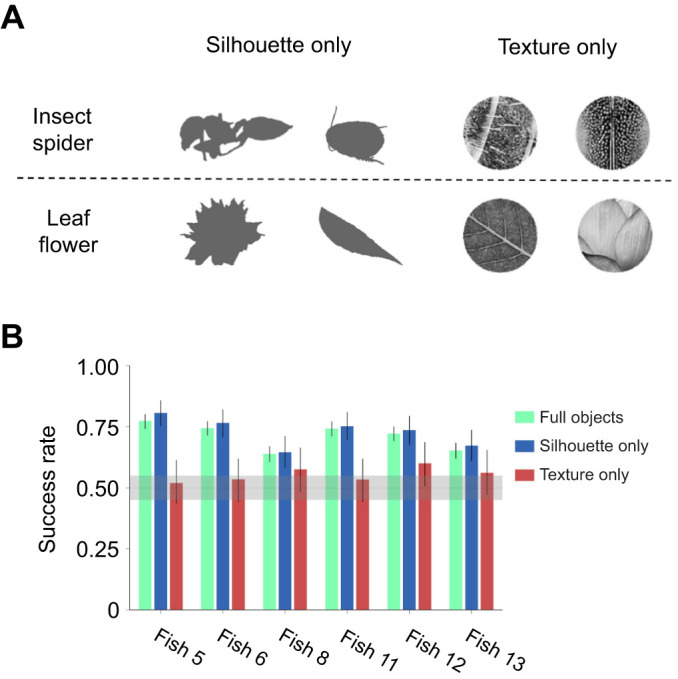
**Shape features are more important to recognition than texture.** (A) Examples of animal target silhouettes and textures (top row) and non-animal target silhouettes and textures (bottom row). (B) Success rate at recognizing the target category in the original experiment with a full object (green bars) and with silhouettes alone (blue bars) and texture alone (red bars) in 6 fish: means±95% HDI. Shading represents a ROPE of 0.05 around the chance level of 0.5. For all fish, no significant difference was found in the response rate between the original and the silhouette experiments: 95% HDI was above the chance level in all fish. In the texture experiment, the 95% HDI range included the chance level of 0.5. For all but two fish, there was a significant difference between the original and the texture experiment. For the shape experiment, we had 200 trials and fish responded in 186–200 trials. For the texture experiment, we had 100 trials and fish responded in 87–100 trials.

To further test the prediction that shape features were more important than texture, we assessed the ability of the fish to perform object recognition after removing all textures and leaving only the silhouette of the image versus removing all the shape information and leaving only texture (Fig. 6A). The experimental procedure was identical to that used in the original categorization experiment.

We found that the fish were able to perform object discrimination between animals and foliage when provided only with the shape but failed to do so when provided only with texture (Fig. 6B). This fact, a finding in itself, also increases our confidence in interpreting results from the model.

### Execution noise drives most fish errors

Allowing the SVM classifier to learn from the fish behavioral data enabled it to perform this categorization task at nearly perfect performance level. Our model (Fig. 5A) attributed the fish errors either to poor classification or to execution noise that was independent of the images. The red line in Fig. 5D shows the results of adding execution noise to decisions based on the model's classification. Inspection shows that it matches the performance of the fish closely.

We next conducted a stringent test involving the re-examination of image pairs. It was premised on the assumption that if the internal image processing mechanism has near-perfect performance, most errors are the result of execution noise. We predicted that there would be no significant difference between the success rates of the fish on previously successful and unsuccessful image pairs.

To test this supposition, we repeated the original categorization experiments with four different sets of images: (1) the image pairs that the fish identified correctly; (2) the image pairs that the fish identified incorrectly; (3) the image pairs labelled correctly by the model trained on the results of each specific fish; and (d) the image pairs that the model labeled incorrectly.

**Fig. 7.**
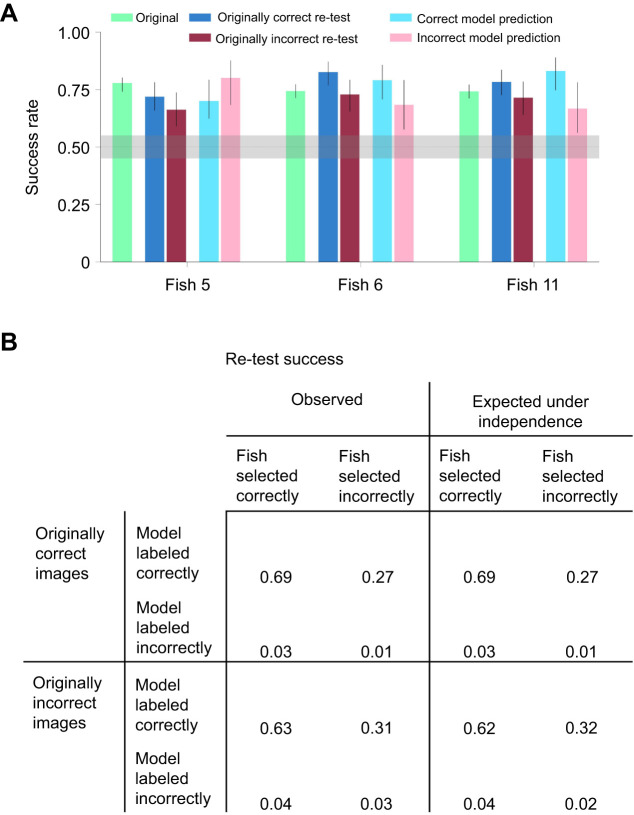
**Fish errors are not correlated with object identity.** (A) The original experiment in object categorization was repeated for selected sets of objects: objects that were previously selected correctly by the fish, objects that were previously selected incorrectly by the fish, objects that the model labeled correctly and objects that the model labeled incorrectly (means±95% HDI). The 95% HDI of the fish success rate for all sets of objects was above chance level for all three fish that finished all sets. (B) Left: portion of images identified correctly and incorrectly by the fish and by the fish-trained model from two datasets: the dataset of images selected correctly in the original categorization task by the fish (top rows) and the set of images selected incorrectly by the fish (bottom rows). Right: success rate for the same groups expected under independence. For the model correct and originally correct conditions, there were 200 trials and fish responded in 192–200 trials. For the originally incorrect condition, there were 140 trials, and fish responded in 136–140 trials. For the model incorrect condition, there were 30 trials and fish responded in 29–30 trials.

The lower bounds of the 95% HDI of the success rate for all fish and all types of targets were well above chance level (Fig. 7A), suggesting that at least part of the errors that the fish made were due to execution noise and not to the inability of the fish object recognition algorithm to identify an object.

In addition, we compared the selection by the fish and the model for two sets of images: images that the fish identified correctly in the original experiment and images that the fish identified incorrectly. For each image in the two sets, there were four possible outcomes: both the fish and the model identified it correctly, the fish was correct and the model was incorrect, the fish was incorrect and the model was correct, and both the fish and the model were incorrect. The success rate for all these possibilities did not differ from the success rate expected under independence (Fig. 7B).

## DISCUSSION

This study explored object recognition of natural objects in the archerfish. At its core, visual object recognition binds the stimulus to an internal representation of visual entities that is invariant to most aspects of the stimulus except object identity (DiCarlo and Cox, 2007). This includes invariance to size, contrast, rotation, viewpoint and illumination, to name only a few, whose variations result in an infinite number of possible projections of the object onto the retina. Our results indicate that the archerfish exhibits this visual function with high accuracy, even when the objects are ecologically relevant and are subject to many visual transformations.

Another important and possibly higher-level feature of object recognition is the ability to categorize objects by generalizing from examples. We tested this ability in the archerfish by training fish on non-repeating sequences of object images from different classes and confronting them with novel stimuli that still belonged to the trained classes (as judged by humans). The archerfish were indeed able to generalize across a wide range of possible objects and successfully perform the task. It is worth mentioning that a similar categorization was studied in sharks, which were able to distinguish between fish and snails (Schluessel and Düngen, 2015). Our study is similar but extends the analysis to provide understanding of which features are important for the animal decision process.

Moreover, we were able to extend this result further with additional novelty of our study: analysis which indicates possible computation done in the fish visual system. For this purpose, we analyzed fish behavior using a model that aimed to replicate the behavior. The model was built as a three-stage cascade composed of visual feature extraction, classification with a learned classifier and incorporation of additive execution noise before the final decision was made. When we trained the classifier based on the selection made by the fish, we found that it achieved almost perfect performance in predicting the true labels of the objects. Furthermore, it exhibited a hierarchy between features (Fig. 5D), suggesting that the fish attributed more importance to shape features than to texture features. We tested this hypothesis with additional behavioral experiments and confirmed it.

The novelty of our analysis is based on the ability to break down the computational complexity of object recognition in the archerfish. While objects are extremely complex, using the SVM-based model we were able to consider many object features simultaneously. Using the model, we were able to sort them and distinguish between significant and non-significant features. Previous studies in object recognition compared the importance of object shape and texture in the recognition process. In primates, shape proved to be more significant than texture (Biederman and Bar, 1999; Hauser, 1997; Rosch et al., 1976), while pigeons were more affected by changes in surface texture (Gibson et al., 2007; Soto and Wasserman, 2014). Our study thus supports a conclusion that the archerfish is more similar to primates in this respect.

### The importance of execution noise in archerfish processing

The model also supports the hypothesis that behavioral classification errors were mainly due to execution noise and were not image specific. According to our model, SVM classifier imitates internal computations. When trained on fish behavioral outcome, it showed significantly higher success rate than did the fish themselves. Adding noise to the output of the classifier produced results similar to the observed behavior (Fig. 5A,D). The gap between the success rate of a classifier and the behavior might be attributed to the execution noise.

Noise is present in every stage of sensory information processing and movement execution (Faisal et al., 2008; Van Beers et al., 2004; van der Vliet et al., 2018). The errors the fish make do not depend on specific images, as we can see from the result of the re-test experiments (Fig. 7A). Thus, our results might indicate that the role of processing noise is minimal, and most of the errors in fish behavioral choices are due to execution noise.

### The neural basis of object recognition in archerfish

Studies suggest that information processing underlying object recognition in the mammalian brain is organized hierarchically and is anatomically located in the ventral stream of the visual cortex (Bracci et al., 2017; Felleman and Van Essen, 1991; Grill-Spector et al., 2001). A visual signal is transferred from the retina to the primary visual cortex V1, where basic features such as oriented lines and edges are extracted (Felleman and Van Essen, 1991; Rust et al., 2005). Information is then transferred through several cortical areas, which select for combinations of visual features, such as orientation and spatial frequency, as well as for higher level geometric features such as curvature (Hegde and Van Essen, 2000). Further downstream, neurons in the inferior temporal cortex have been reported to process complex object features and be tuned to specific object classes such as faces or body parts (Cadieu et al., 2007; Fujita, 2002; Gallant et al., 1996; Lehky and Tanaka, 2016).

Less information is available on visual processing in the archerfish. Previous work on the archerfish has examined visual neural processing in the retina (Segev et al., 2007) and in the optic tectum (Ben-Tov, et al., 2013; Ben-Tov et al., 2015), the latter being the largest visual processing area in the archerfish brain (Karoubi et al., 2016). The archerfish optic tectum contains processing stages similar to those found in the early visual system of mammals (Reichenthal et al., 2018). However, it remains unclear whether this area is also the main brain region responsible for object recognition in the archerfish or whether other regions, perhaps within the telencephalon, provide critical functions toward that end.

### Previous studies of object recognition in archerfish and other fish

One of the most seminal studies on object recognition in archerfish focused on human face recognition (Newport et al., 2016, 2018). The findings showed that archerfish could be trained to recognize human faces in that the fish correctly discriminated one specific face from others, though with apparent difficulty as accuracy decreased markedly on rotated versions of the same face. By contrast, our results show that the fish could identify the same object despite various deformations, including rotation. This could be due to the improved recognition capacity related to the stimuli we used, which were chosen for their ecological relevance (recall that we used insects and foliage). Alternatively, it could also be because the face images were much more difficult to discriminate because of the similarity between images.

It should be noted that we did not address the importance of features for specific types of stimuli. For example, discriminating spiders from all the other objects might require the use of different features and might explain why overall shape is the most useful cue in this particular context. Other studies have examined the ability of archerfish to recognize simple shapes to test various forms of fish visual behavior, including visual search (Ben-Tov et al., 2015; Reichenthal et al., 2019; Reichenthal et al., 2020), symbol–value association and discrimination (Karoubi et al., 2017) as well as the generalization of the abstract concept of same and different (Newport et al., 2014, 2015). The current study is nevertheless the first to reveal ecologically relevant object recognition in this species.

An additional important study of object recognition in teleosts was performed in the goldfish, where fish were able to identify a handful of single objects irrespective of rotation (DeLong et al., 2018). Our study extends this finding by showing that teleostei are able to categorize and overcome transformation of translation, rotation, size (or taken together, what is known as similarity transformations) and contrast.

### Considerations in modeling fish behavior and limitations

To assess the influence of the specific classifier, we tested several other classifiers including *k*-nearest neighbor, discriminant analysis and neural networks. The success rates of these classifiers were similar to those observed using the SVM both for predicting image true labels and for predicting fish behavior (Fig. 5C). Therefore, the choice of the classifier did not appear to significantly affect the results.

In addition, naive application of the SVM on the raw images, by trying to directly reverse-engineer the image features used by the fish, failed. This is probably due to the high dimensionality of the problem at hand (Afraz et al., 2014). For this reason, we implemented a feature extraction approach followed by application of the SVM, which is the standard approach in the field (Brunelli and Poggio, 1993; Chandra and Bedi, 2018). Finally, it should be noted that our findings do not imply that the neural computations underlying object recognition in the archerfish actually employ an identical or similar algorithm to the one generated by our model.

### Conclusions

We examined the ability of archerfish to recognize ecologically relevant objects. Using a model for fish selection, we showed which visual features were used by the archerfish during visual processing. Future studies should explore whether and how these visual features are represented and used in the neural circuitry responsible for object recognition in archerfish.
